# Imaging features of intra-abdominal and intra-pelvic causes of hirsutism

**DOI:** 10.1007/s00261-024-04189-9

**Published:** 2024-03-19

**Authors:** Arleen Li, Noah Bloomgarden, Shari Friedman, Milana Flusberg, Victoria Chernyak, Robert Berkenblit

**Affiliations:** 1 Department of Radiology, Montefiore Medical Center, Bronx, NY USA; 2 Department of Endocrinology, Montefiore Medical Center, Bronx, NY USA; 3 Department of Radiology, Westchester Medical Center, Valhalla, NY USA; 4 Department of Radiology, Columbia University Medical Center, New York, NY USA; 5 Department of Radiology, Memorial Sloan Kettering Cancer Center, New York, NY USA

**Keywords:** Hirsutism, Adrenal, Ovarian, Hyperandrogenism, Imaging features

## Abstract

Hirsutism is a relatively common disorder which affects approximately 5% to 15% of women. It is defined by excessive growth of terminal hair in women, which primarily affects areas dependent on androgens, such as the face, abdomen, buttocks, and thighs. Hirsutism can be caused by a variety of etiologies, which are most often not lifethreatening. However, in some cases, hirsutism can be an indicator of more serious underlying pathology, such as a neoplasm, which may require further elucidation with imaging. Within the abdomen and pelvis, adrenal and ovarian pathologies are the primary consideration. The goal of this manuscript is to review the etiologies and imaging features of various intra-abdominal and intra-pelvic causes of hirsutism.

## Introduction

Hirsutism is a relatively common disorder which affects approximately 5% to 15% of women [1]. It is defined by excessive growth of terminal hair in women, which primarily affects areas dependent on androgens, such as the face, abdominal wall, buttocks, and thighs. Hirsutism can be caused by a variety of etiologies, including adrenal and ovarian causes. In most cases, the underlying condition is not life-threatening, however, in some cases hirsutism can be an indicator of more serious underlying pathology, such as a neoplasm. Hirsutism may also cause psychological distress in some women. The underlying cause of hirsutism often cannot be established based on clinical exam but is often elucidated based upon biochemical assessment followed by imaging [1, 2]. This manuscript reviews the etiologies and imaging characteristics of various intra-abdominal and intra-pelvic (i.e., adrenal and ovarian) causes of hirsutism.

## Pathophysiology and clinical evaluation

Hirsutism results from increased exposure and/or sensitivity to androgens at the level of the hair follicle, leading to transformation from thin vellus to thick terminal fibers. The most common sources of increased endogenous androgen levels in women with hirsutism are the ovaries and adrenal glands [2, 3]. While the ovaries are a major source of testosterone, the adrenal glands are a primary source of dehydroepiandrosterone sulfate (DHEAS) and androstenedione, which are weaker androgens [4].

The clinical evaluation and work-up for hirsutism begins with a detailed history and physical exam. True hirsutism should be distinguished from hypertrichosis, a generalized increase in hair growth throughout the body [1]. A modified Ferriman-Gallwey score, which scores the degree of hirsutism in nine androgen-dependent areas on the body, can be used to help determine the severity of hirsutism. However, this method is limited by cosmetic procedures and its overall subjectivity. Additionally, the presence or absence of symptoms of virilization should be noted. Virilization is most commonly recognized by the development of phenotypically male characteristics in a female, which may include voice deepening, loss of menstruation, and clitoromegaly. Note should also be made of any medications associated with hirsutism, such as anabolic steroids, exogenous testosterone, danazol, and oral contraceptives containing levonogestrel, norethindrone, or norgestrel. Family history of hirsutism is also important, as 50% of women with hirsutism have a positive family history. Patient distress related to the degree of hirsutism should also be considered [1, 3, 5].

Ovarian or adrenal androgen-secreting tumors are frequently associated with virilization, rapid progression of hirsutism, and cessation of menstruation. Features of hyperandrogenism which occurs outside of the menarcheal period should raise the suspicion for the possibility of an ovarian or adrenal tumor. Patients suspected to have a malignancy as an underlying cause for hirsutism should undergo comprehensive laboratory testing, including assessment of serum testosterone, 17-hydroxyprogesterone (17-OHP), DHEAS and androstenedione. On the other hand, patients who present with peripubertal onset hirsutism with slow progression, preservation of regular menses, and otherwise normal physical exam have a low likelihood of having an underlying neoplasm, though still require hormonal evaluation [1–4].

In the rare circumstance when both ovarian and adrenal pathology are seen on imaging, further laboratory testing such as adrenal and/or ovarian vein sampling may be needed to confirm the source of hyperandrogenism [4, 6].

## Adrenal causes of hirsutism

Adrenal pathologies causing hirsutism are rare but can have serious consequences for the patient. The adrenal glands are composed of two separate functional components: the cortex and medulla. The adrenal cortex is responsible for production of corticosteroids, aldosterone, and adrenal androgens while the medulla generates catecholamines [7].

Of the many adrenal pathologies that can be seen on imaging, only a few are associated with hirsutism, including adrenocortical carcinoma, congenital adrenal hyperplasia, non-classic congenital adrenal hyperplasia, and androgen-secreting adrenal adenoma. Another differential consideration for hirsutism is Cushing’s syndrome, which can be caused by increased adrenocorticotropic (ACTH) levels either secreted by the pituitary gland or arising from an ectopic source, or due to increased corticosteroid secretion from adrenal lesions such as adrenal adenoma or adrenocortical carcinoma [7]. The imaging features of these entities will be described in this section.

### Adrenocortical carcinoma

Adrenocortical carcinoma (ACC) is a rare primary tumor of the adrenal gland, which occurs with equal frequency between men and women, most commonly during the fifth decade of life [7]. Approximately 50% of ACCs are functional, with most presenting with a mixed clinical picture of Cushing’s syndrome and hyperandrogenism [7]. Cases of purely virilizing ACC have also been reported but are relatively uncommon [8].

**Fig. 1:**
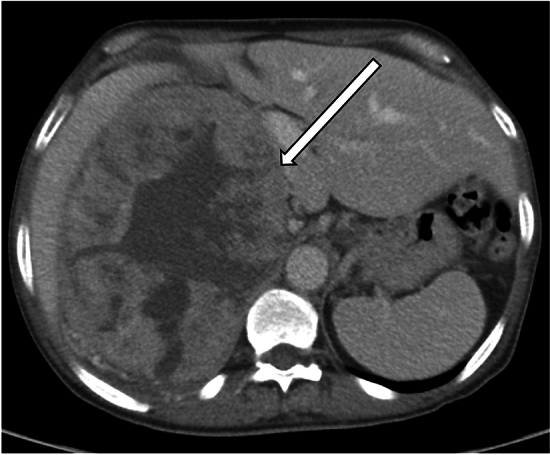
A representative case of ACC in a 64-year-old man with abdominal pain. The axial CT image of the right adrenal gland demonstrated a large necrotic mass (arrow) with associated mass effect on the adjacent viscera. Retroperitoneal lymphadenopathy was present as well (not shown). Pathology was consistent with primary adrenocortical carcinoma, with invasion into the periadrenal/renal adipose tissue

**Fig. 2:**
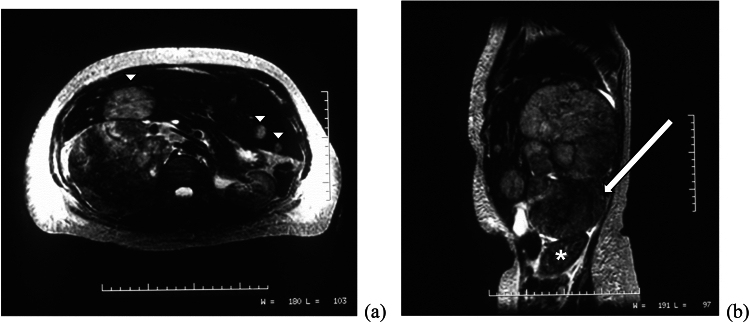
A 60-year-old woman presented with marked hirsutism and elevated androgen levels. Axial (**a**) and sagittal (**b**) T2-weighted MRI images demonstrate a large right adrenal mass (arrow) which displaces the kidney (*) inferiorly. Multiple liver metastases are also present (arrowheads)

On computed tomography (CT) images, ACC typically appears as a large heterogeneous mass with central necrosis, with 30% containing calcifications [7]. Smaller lesions may appear homogeneous on non-contrast enhanced CT [9]. On contrast-enhanced CT, there is typically inhomogeneous enhancement of the tumor, typically with relatively less enhancement centrally due to necrosis (Fig. 1) [9]. Magnetic resonance imaging (MRI) of these tumors often demonstrates heterogeneous signal intensity, typically with hypointense or isointense T1 signal compared to liver parenchyma. However, there may be areas of high T1 signal intensity due to the presence of hemorrhage. On T2-weighted images, ACC typically appears hyperintense to liver parenchyma and is often heterogeneous due to cystic areas and hemorrhage (Fig. 2). On chemical shift imaging, there may be small areas of signal loss due to regions of intracytoplasmic lipid [9]. ACC generally enhances avidly early with slow washout [9]. Compared to CT, MRI is better for identifying extension of the tumor into surrounding structures as well as vascular invasion [7, 9].

### Congenital adrenal hyperplasia

Congenital adrenal hyperplasia (CAH) is a rare autosomal recessive disorder associated with an excess production of androgens. It results from a deficiency in one of five enzymes involved in steroid synthesis in the adrenal glands. The most common enzyme deficiency is that of 21-hydroxylase, followed by that of 11-beta-hydroxylase. Classic CAH presents in the perinatal period with salt wasting and/or features of virilization in male and female infants. The nonclassical variant of the disorder manifests later in life, typically in adolescence or early adulthood, and frequently presents with a phenotype that overlaps with hirsutism and irregular menses associated with polycystic ovarian syndrome (PCOS) [7, 10].

**Fig. 3:**
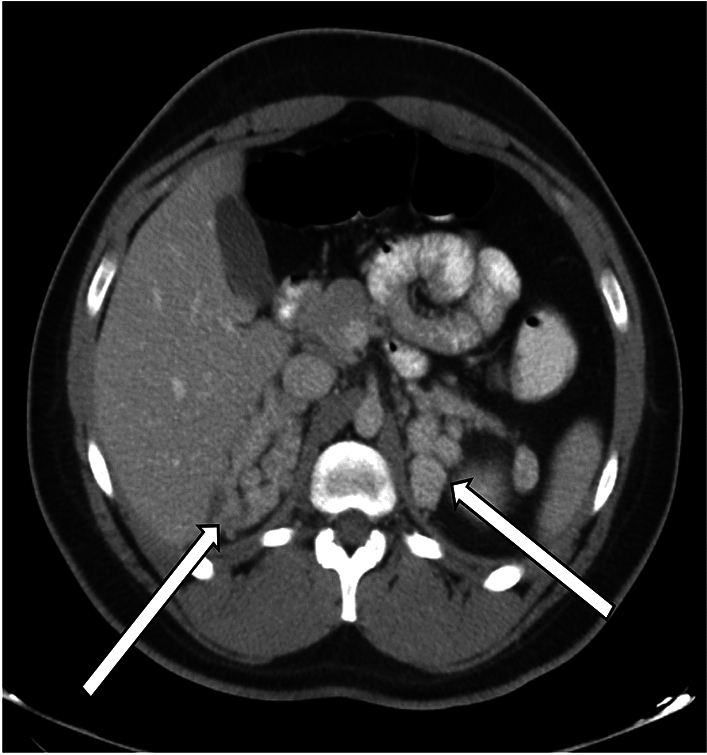
A representative case of CAH in a 21-year-old man, with axial CT showing nodular thickening of both adrenal glands (arrows)

**Fig. 4:**
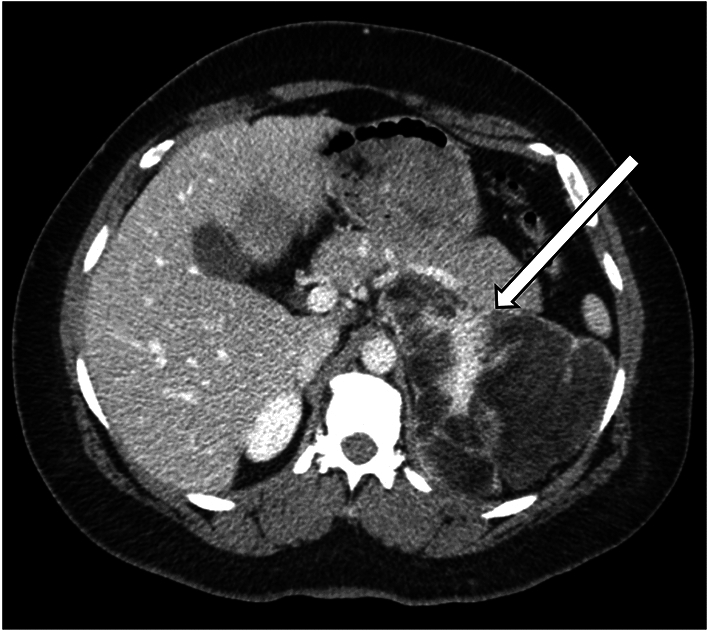
A representative case of adrenal myelolipoma in the setting of CAH in a 42-year-old man with history of ambiguous genitalia who underwent CT for a suspected adrenal mass. CT showed a large left adrenal mass (arrow) with areas of fat attenuation and enhancement, consistent with adrenal myelolipoma. The right adrenal gland had previously been resected due to hemorrhage, with surgical pathology of the right adrenal gland consistent with myelolipoma and diffuse cortical hyperplasia of the adrenal gland

Imaging findings in patients with CAH classically demonstrate thickened, nodular glands bilaterally with relatively homogenous enhancement (Fig. 3). CAH may also be associated with adrenal adenomas and myelolipomas, frequently in the setting of inadequate steroids controlling ACTH stimulation (Fig. 4) [10–13].

## Ovarian causes of hirsutism

Several ovarian pathologies can also manifest with hirsutism, including both benign and malignant entities. These entities may be discovered incidentally or during imaging work-up for hirsutism and virilization. Ovarian lesions are often seen on ultrasound; however, indeterminate lesions can be further evaluated on CT, MRI, and/or positron emission tomography (PET) [14].

### Polycystic ovary syndrome (PCOS)

Polycystic ovary syndrome (PCOS) is the most common cause for hyperandrogenism in premenopausal women [15, 16]. It is characterized by hyperandrogenism, ovarian abnormalities, and numerous ovarian cysts. The exact etiology of PCOS remains unknown, although posited hypotheses include increased frequency of gonadotropin-release hormone pulses stimulating androgen production by theca cells. PCOS is also associated with insulin resistance, leading to an increased risk of type 2 diabetes mellitus and cardiovascular disease [15–17].

**Fig. 5:**
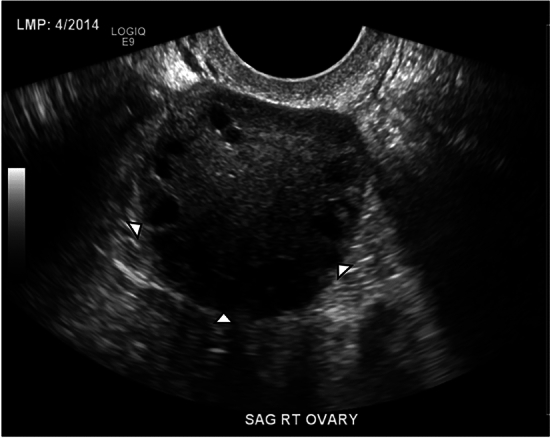
A 28-year-old woman with no significant past medical history presented with irregular menstrual periods. She underwent pelvic ultrasound which demonstrated mildly enlarged ovaries with numerous peripherally oriented small follicles (arrowheads). The right ovary measured 22 mL in volume, while the left ovary measured 16 mL (not shown)

**Fig. 6:**
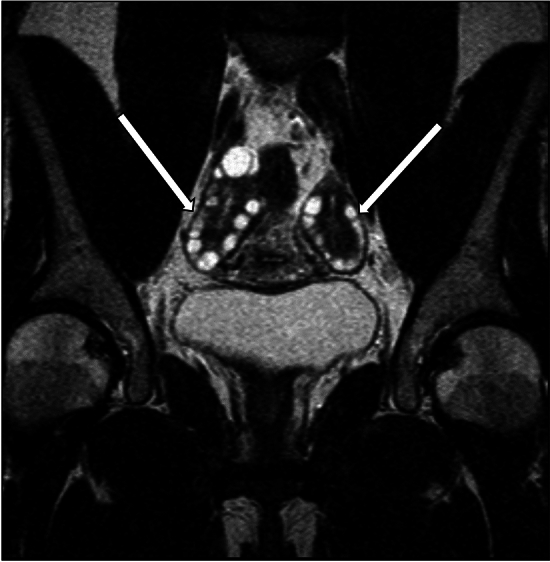
T2-weighted coronal MRI of the pelvis of a 29-year-old woman who presented with hirsutism and right ovarian cyst on ultrasound. Enlargement of both ovaries, which contain numerous peripheral small follicles in a “string of pearls” distribution, is seen (arrows). This appearance is compatible with PCOS

One of the criteria used for diagnosing PCOS is the presence of multiple ovarian cysts, with either 12 or more cysts in one ovary or ovarian volume greater than 10 mL. Follicles are often peripherally oriented in the ovary, classically described as a “string of pearls” appearance (Figs. 5, 6) [16, 17]. However, imaging findings are not necessary for the diagnosis of PCOS if other clinical criteria are met, including biochemical evidence of hyperandrogenism and evidence of ovulation abnormality [16, 17].

### Ovarian hyperthecosis

Ovarian hyperthecosis is characterized by the presence of islands of luteinized theca cells in the ovarian stroma. These luteinized theca cell nests are scattered throughout the ovarian stroma in ovarian hyperthecosis. This is in contrast with PCOS, where they are located primarily in areas around the cystic follicles [18]. The clinical presentation of ovarian hyperthecosis is similar to PCOS, including menstrual dysfunction (which includes postmenopausal bleeding), obesity, insulin resistance, and hyperandrogenism. However, women with hyperthecosis tend to have higher testosterone levels, more hirsutism, and a higher incidence of virilization compared to those with PCOS. Ovarian hyperthecosis most commonly occurs in perimenopausal or postmenopausal women and is the second most common cause of hyperandrogenism in postmenopausal women [18, 19].

**Fig. 7:**
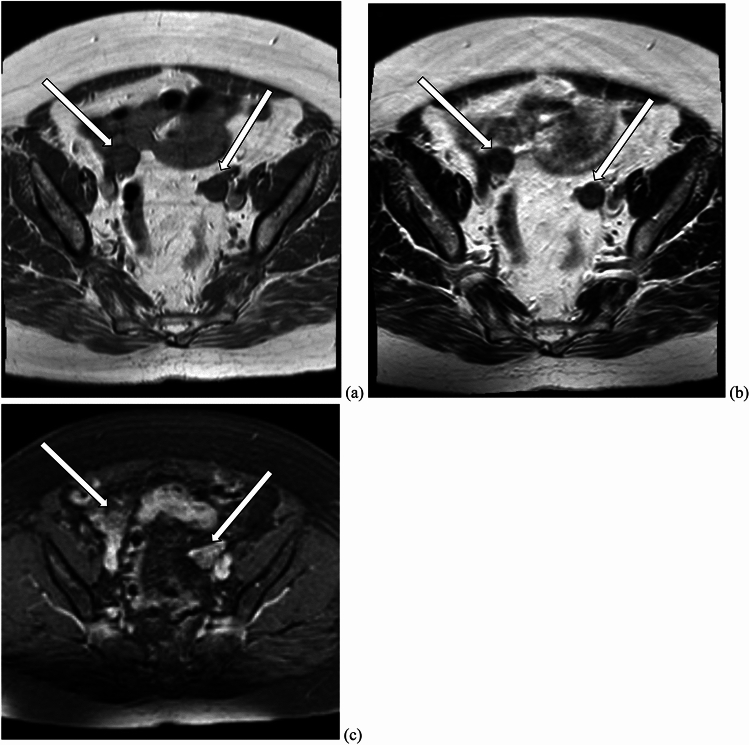
A 69-year-old woman with a history of hypertension, diabetes mellitus, and hepatitis C, who presented with 2 years of hirsutism, with laboratory results demonstrating elevated total testosterone levels and low DHEAS level. MRI abdomen demonstrated stable mild thickening of the left adrenal gland and normal right adrenal gland. Subsequent pelvic MRI was significant for enlargement of both ovaries (arrows) with low to intermediate signal on T1-weighted sequences (image **a**). On T2-weighted images, the ovaries demonstrated relatively decreased signal intensity (image **b**). Contrast-enhanced images (image **c**) demonstrated mild enhancement of the ovaries. The decision was made for the patient to undergo bilateral salpingo-oophorectomy. Final pathology was consistent with ovarian stromal hyperplasia and stromal hyperthecosis

Ultrasound findings of ovarian hyperthecosis are nonspecific and may show an increase in ovarian size, often involving both ovaries. There are typically no focal areas of increased vascularity on color Doppler. The increased ovarian stroma in hyperthecosis tends to gently push normal-appearing follicles towards the periphery, in contrast with PCOS, which tends to present with numerous small follicles with thickened walls in a setting of relatively normal ovarian stroma [20]. However, failure to detect an ovarian lesion on pelvic ultrasound does not exclude hyperthecosis. MRI may be helpful for further evaluation in patients with possible ovarian hyperthecosis, although its role in diagnosing this entity is not fully established. The described MRI appearance of ovarian hyperthecosis have included symmetric bilateral ovarian enlargement with homogeneous T2 hypointensity of the ovaries, as well as mild enhancement (Fig. 7). Rarely, this entity can manifest as bilateral ovarian masses which are isointense to uterine myometrium on T1- and T2-weighted images [19, 21].

Treatment of ovarian hyperthecosis includes medical management such as gonadal suppression and surgical management (oophorectomy or ovarian wedge resection) [18].

### Fibrothecoma

Fibrothecomas, fibromas, and thecomas are benign solid ovarian sex-cord stromal tumors. They are relatively uncommon, making up around 4% of all ovarian tumors [22]. While fibromas consist of benign fibroblast and collagen bundles arranged in whorls, thecomas are made of theca cells containing cytoplasmic lipid with varying degrees of fibrosis. The term ‘fibrothecoma’ reflects the frequently noted histologic overlap of these lesions. Fibrothecomas can occasionally be hormonally active. Meigs syndrome is a clinical triad consisting of fibroma, ascites, and pleural effusion, which resolves after resection of the fibroma. Fibromas and fibrothecomas can also present in association with basal cell nevus (Gorlin-Goltz) syndrome, which consists of large multinodular ovarian fibromas, multiple basal cell carcinomas of the skin, odontogenic keratocysts, and other findings [15, 23, 24].

**Fig. 8:**
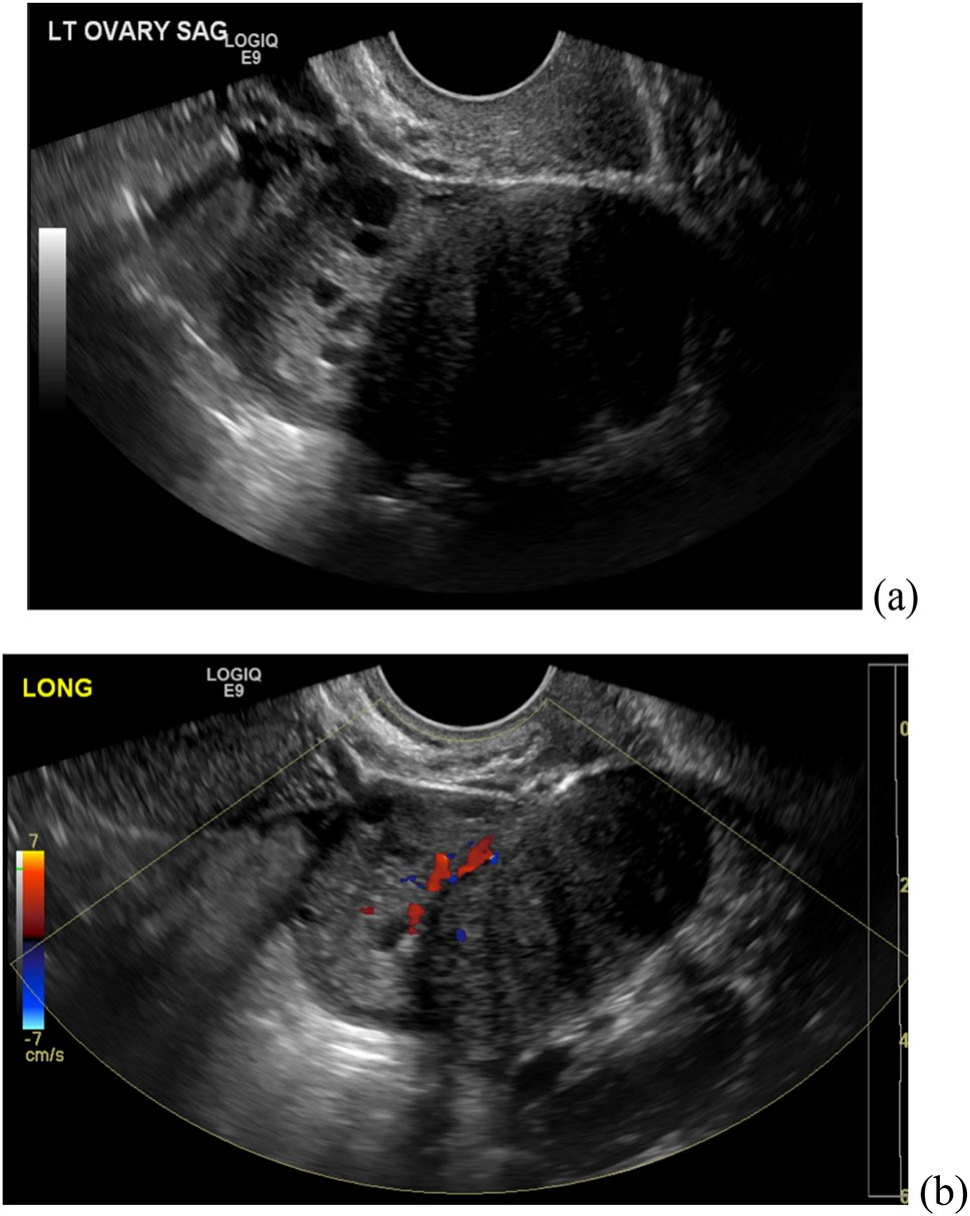
A 21-year-old woman with no significant past medical history, who presented with menstrual irregularity and pelvic pain. Pelvic ultrasound demonstrated a solid left ovarian mass (image **a**) with internal vascular flow (image **b**), most consistent with a solid ovarian lesion such as fibrothecoma

**Fig. 9:**
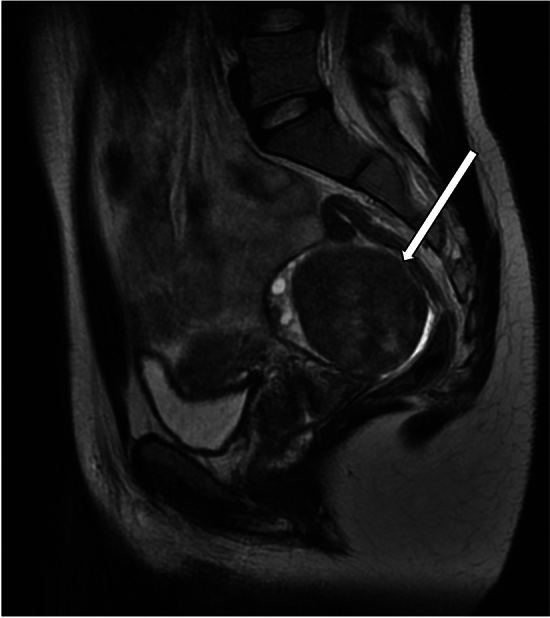
Pelvic MRI in the same patient shows a solid left ovarian lesion with low to intermediate T2 signal intensity (arrow), as well as mild uniform enhancement, consistent with fibrous tumor such as fibrothecoma. The patient underwent left salpingo-oophorectomy, with final pathology consistent with ovarian fibrothecoma

Ultrasound findings of ovarian fibrothecoma are often nonspecific, typically a hypoechoic solid ovarian mass (Fig. 8), although hyperechoic masses have also been reported [25]. On CT imaging, most fibrothecomas present as solid adnexal masses with variable enhancement, although many demonstrate delayed homogeneous enhancement [24, 25]. On MRI, the solid portions of the tumor typically demonstrate homogeneous low signal compared to myometrium on T1- and T2-weighted images, with mild enhancement on contrast-enhanced images (Fig. 9). Associated edema and cystic degeneration are commonly seen, especially in large fibrothecomas [23, 25]. Calcifications and hemorrhage are rare. The majority of these lesions are unilateral and can rarely be malignant [23–25].

Although functional ovarian fibrothecoma are rare, there have been several reported cases of ovarian thecomas causing hirsutism, menstrual irregularity and/or amenorrhea. The mainstay treatment of hyperandrogenic ovarian thecomas is complete surgical excision [26].

### Leydig cell tumor

Leydig cell tumors are rare ovarian sex-cord stromal tumors, accounting for less than 0.1% of all ovarian tumors [25]. They primarily occur in postmenopausal women and are typically benign and unilateral. These tumors are functional and typically produce testosterone, leading to hyperandrogenism and virilization. Histological characteristics include nodules of Leydig cells with neoplastic stromal proliferation. Approximately half of patients develop signs of virilization, while another third present with estrogenic manifestations, which can be secondary to direct estrogen secretion from the tumor, peripheral conversion of androgen to estrogen, associated ovarian stromal hyperthecosis, or a combination of factors [27]. The mainstay treatment is surgical resection, especially given the difficulty in distinguishing benign versus malignant variants of this tumor based on clinical presentation and imaging alone. In some patients who may not be good surgical candidates, medical hormone treatment (e.g., gonadotropin releasing hormone agonists) may be a viable management option [25, 27].

**Fig. 10:**
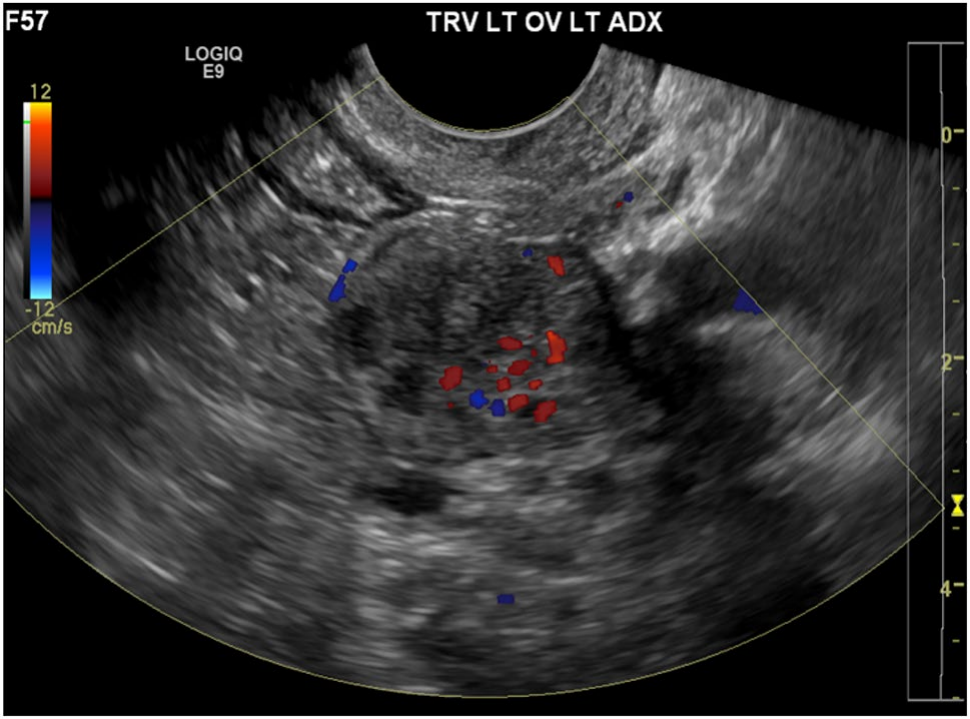
A 57-year-old woman underwent pelvic ultrasound exam showing a solid left ovarian mass with internal vascularity on Doppler ultrasound exam

**Fig. 11:**
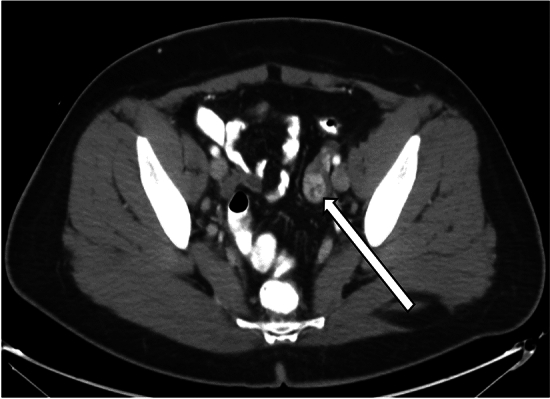
The patient subsequently underwent contrast-enhanced CT examination, which demonstrated a corresponding heterogeneously enhancing left ovarian lesion (arrow). Final pathology confirmed Leydig cell tumor

**Fig. 12:**
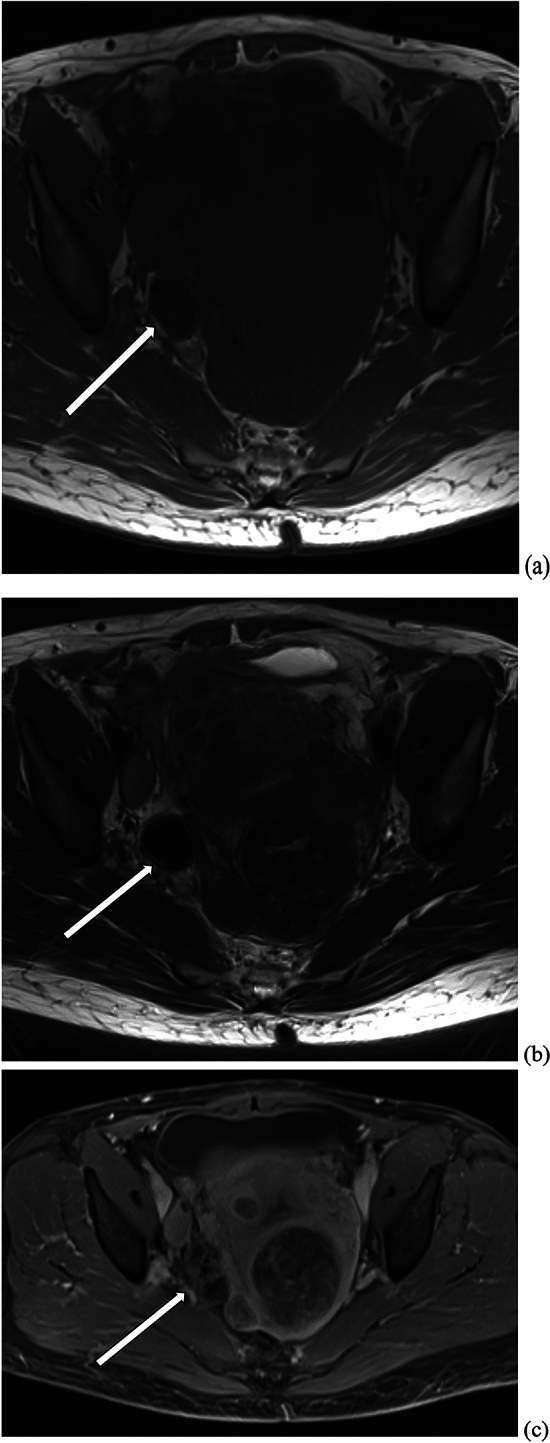
A 68-year-old woman with a past medical history of hypertension and uterine fibroids presented with a 3-year history of hirsutism, with increased hair on shoulders. Laboratory results showed elevated testosterone, suppressed luteinizing hormone (LH) and follicle-stimulating hormone (FSH), and low DHEAS levels. Pelvic MRI showed a right ovarian lesion (arrow) with low to intermediate T1 signal intensity (image **a**), low T2 signal intensity (image **b**), and heterogeneous enhancement (image **c**). On surgical resection, a 2.5 cm right ovarian mass was noted, with final pathology consistent with benign Sertoli–Leydig cell tumor. On follow up visits, the patient reported improving hirsutism and had decreasing testosterone levels

On ultrasound, Leydig cell tumors are typically unilateral and tend to be isoechoic to myometrium (Fig. 10) [25]. On CT, these tumors tend to present as a hypodense solid ovarian mass (Fig. 11) [25]. MRI characteristics of Leydig cell tumors include variable T2-weighted signal depending on the amount of fibrous content (Fig. 12) [25]. Given their small size, they can be difficult to differentiate from normal ovarian tissue. Diffusion weighted sequences can be helpful in these cases, as the tumor tends to demonstrate slightly higher signal intensity on these sequences compared to normal ovary [25]. These tumors demonstrate gradually increasing and delayed enhancement [25].

Other sex-cord stromal tumors, such as granulosa cell tumors and steroid cell tumors, have also been reported in association with virilization and hirsutism, although these cases are extremely rare [28, 29].

### Androgen-secreting mature cystic teratoma

Mature cystic teratomas, also commonly referred to as dermoids, represent approximately 10–20% of ovarian neoplasms and are commonly seen in reproductive age women [30]. They consist of well-differentiated ectodermal, endodermal, and mesodermal germ cell layers. While most ovarian teratomas are nonfunctional and usually discovered incidentally, rarely teratomas can secrete androgens, leading to virilization and hirsutism. Cases of single or multiple ovarian teratomas in association with polycystic ovaries have been reported [31]. Polycystic ovaries can be seen secondary to high circulating androgens. However, there have also been cases of hirsutism associated with ovarian teratomas without the presence of polycystic ovaries. In some of these lesions, histologic evaluation demonstrated luteinized cells within the stroma of the tumor, which may have been the cause for hyperandrogenism [15, 30, 31].

**Fig. 13:**
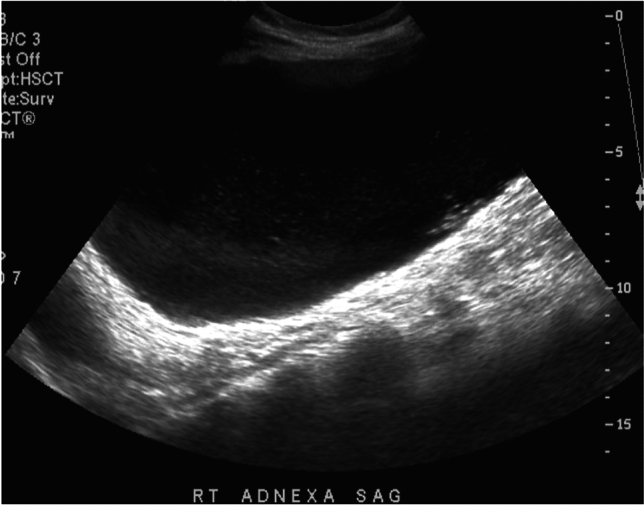
A 15-year-old female with a past medical history of epilepsy presented with hirsutism and elevated testosterone levels. Pelvic ultrasound demonstrated a large cystic structure in the right adnexum containing internal low-level echoes

**Fig. 14:**
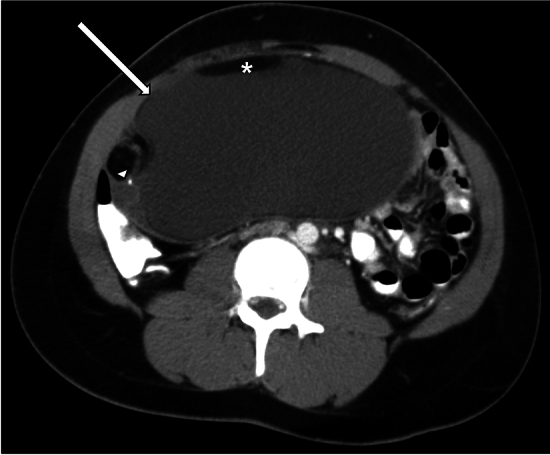
Contrast-enhanced CT of the pelvis in the same patient as Fig. 13, which demonstrates a large fluid-filled cystic structure (arrow) with adjacent solid nodule (arrowhead) containing punctate calcification. Macroscopic fat (*) is present anteriorly in the mass. Final pathology was consistent with mature teratoma. The patient’s testosterone levels normalized after surgery

Several ultrasound features of ovarian mature cystic teratomas have been described. A classic appearance is that of a cystic adnexal lesion (Fig. 13) with a densely hyperechoic nodule, known as a Rokitansky nodule, projecting into the lumen of the cyst. Other described ultrasound appearances include a homogeneously hyperechoic lesion, fat-fluid levels, dermoid mesh (linear echogenic bands floating within the cyst, representing hair fibers), and the ‘tip of the iceberg sign’ which describes an echogenic mass containing hair causing posterior acoustic shadowing [32–34]. CT findings of ovarian teratomas also typically include a unilocular cyst with a solid Rokitansky nodule which contains fat and hair (Fig. 14). Calcification of the cyst wall may be present or absent [33, 34]. On MRI, the macroscopic fat-containing portion of the dermoid follows the expected signal intensity of fat on all sequences [33, 34].

## Conclusion

Hirsutism, especially when rapidly progressive and/or associated with symptoms of virilization, can be a sign of hyperandrogenism, which may be secondary to ovarian or adrenal etiology. Thorough clinical history and physical exam as well as an assessment of signs of virilization are helpful to differentiate severity and acuity of the patient’s underlying pathology. Hirsutism occurring outside the menarcheal period should raise suspicion for an underlying process. Although many intra-abdominal causes of hirsutism are benign, some can be malignant, thus, further investigation with imaging may be warranted. Of the intra-abdominal pathologies associated with hirsutism, ovarian pathologies are more common, including PCOS, ovarian hyperthecosis, fibrothecoma, Leydig cell tumor, and androgen-secreting cystic teratoma. Adrenal causes are less common but include ACC, CAH, and hypercortisolism. Understanding the spectrum of disease pathology in patients with hirsutism will help radiologists provide value in the care of these patients.
